# Sex disparities in dementia mortality across the American continent: temporal trends

**DOI:** 10.1590/1980-5764-DN-2025-0306

**Published:** 2025-09-29

**Authors:** Lucas Casagrande Passoni Lopes

**Affiliations:** 1 Universidade de São Paulo, Faculdade de Medicina de Bauru, Bauru SP, Brazil.

**Keywords:** Dementia, America, Mortality, Time, Sex, Demência, América, Mortalidade, Tempo, Sexo

## Abstract

Dementia has emerged as a growing global health concern, particularly in aging populations across the Americas. Despite increasing recognition of its burden, substantial gaps remain in understanding its mortality patterns, especially regarding sex differences and regional disparities.

**Objective:**

To evaluate the temporal trends of sex disparities in dementia mortality across the American Continent.

**Methods:**

This ecological study used Pan American Health Organization mortality data (2000–2019) validated against national and World Health Organization sources. Age-adjusted dementia mortality rates by sex and country were analyzed using joinpoint regression to estimate trends and detect significant temporal changes. Results were interpreted using annual percentage changes and their confidence intervals to classify trends as increasing, decreasing, or stationary.

**Results:**

Across the Americas, dementia mortality trends were highly variable by country and sex. Some nations saw parallel trends between men and women, while others exhibited sex-based divergence in direction or intensity. Despite this variability, rising mortality was a common pattern in many locations, especially in North America and parts of South America.

**Conclusion:**

Dementia mortality trends across the Americas reveal marked sex differences and regional heterogeneity, reflecting a complex interaction of demographic, social, and health system factors. These findings emphasize the need for gender-sensitive public health strategies, improved surveillance, and cross-national research to inform equitable and effective dementia care policies.

## RESUMO.

As demências são uma crescente preocupação global de saúde, particularmente entre as populações idosas nas Américas. Apesar do crescente reconhecimento de sua importância, ainda existem lacunas substanciais na compreensão de seus padrões de mortalidade, especialmente com relação às diferenças entre os sexos e disparidades regionais.

**Objetivo:**

Avaliar as tendências temporais das disparidades entre os sexos na mortalidade por demência ao longo do continente americano.

**Métodos:**

Este estudo ecológico utilizou dados de mortalidade da Organização Pan-Americana de Saúde (2000–2019) validados com base em fontes nacionais e da Organização Mundial da Saúde. As taxas de mortalidade por demência ajustadas por idade, sexo e país foram analisadas usando regressão joinpoint para estimar tendências e detectar mudanças temporais significativas. Os resultados foram interpretados usando annual percentage changes e seus intervalos de confiança para classificar as tendências como crescentes, decrescentes ou estacionárias.

**Resultados:**

As tendências de mortalidade por demência foram altamente variáveis por país e sexo. Algumas nações apresentaram tendências paralelas entre homens e mulheres, enquanto outras exibiram divergências em direção ou intensidade baseadas no sexo. Apesar dessa variabilidade, o aumento da mortalidade foi um padrão comum em muitos locais, especialmente na América do Norte e em partes da América do Sul.

**Conclusão:**

As tendências de mortalidade por demência nas Américas revelam diferenças marcantes entre os sexos e heterogeneidade regional, refletindo uma interação complexa de fatores demográficos, sociais e do sistema de saúde. Esses achados enfatizam a necessidade de estratégias de saúde pública com perspectiva de gênero, vigilância aprimorada e pesquisas transnacionais para subsidiar políticas de tratamento da demência equitativas e eficazes.

## INTRODUCTION

 Dementia is a neurological syndrome characterized by the deterioration of cognitive domains that encompasses a broad spectrum of pathologies, often accompanied by behavioral and psychological symptoms^1,2^. Its pathophysiology is complex and multifactorial, including diverse pathological pathwaysl^2^. Sex-based differences in dementia incidence and mortality are increasingly recognized, but remain a debatable issue, especially in regions like the Americas^3,4^. 

 Globally, dementia is emerging as a major public health challenge^5^. With rising life expectancy and aging populations, particularly in low- and middle-income countries, the number of people living with dementia in 2019 was roughly 57 million individuals and is projected to surpass 150 million by 2050^5^. Despite this dramatic rise, significant gaps remain in diagnosis, care access, and health policy responses, especially in regions with limited resources^5^. The economic and social burdens are considerable, including healthcare costs, caregiver strain, and productivity losses^5^. 

 In the Americas, demographic transitions and unequal access to health services create unique epidemiological contexts for dementia^6^. For instance, some studies report that the trends in individuals with more than 65 years of age are as high as in high-income countries, while the prevalence in Latin American individuals aged between 65 and 69 is higher than in European individuals^6^. 

 Yet, reliable data on dementia-related mortality, especially disaggregated by sex, remain limited or inconsistently reported across many countries in the region^6^. While the Pan American Health Organization (PAHO) has prioritized the monitoring of dementia and related non-communicable diseases, transforming raw mortality data into accessible, analyzable trends remains a key challenge^6^. Thereto, this study was developed to evaluate the temporal trends in dementia mortality across the American continent, comparing its patterns between sexes. 

## METHODS

### Study design

 This is a retrospective, population-based ecological study designed to analyze sex disparities in dementia-related mortality trends across countries in the American continent. 

### Data source

 Data employed in this research were retrieved from PAHOs’ records, through its official online platform (https://www.paho.org/en). Aiming to ensure data reliability, data were cross-referenced with national health statistics published by individual countries when available, as well as with WHO’s Global Health Observatory data. 

### Data collection

 Data were mannualy collected from the PAHO’s archives, and they were transposed into Google Drive spreadsheet ®. Data were stratified by its origin country, individual’s sex, reported year, and the age-adjusted dementia mortality number per 100,000 individuals. Only American countries with complete data on dementia mortality reported betwixt 2000 and 2019, the longest time range in PAHO’s archives on the referred issue, were included. 

### Data analysis

 Data analysis was made by using the Joinpoint software 5.4.0. In case the software calculates a linear logarithmic trend of the evaluated variable (Dementia age-adjusted mortality rates) over time (2000 - 2019). Based on this calculation, the software identifies deviations (inflexion points) that indicate patterns of increase, decrease, or stability in the evaluated trends. The analysis is interpreted by identifying the Annual Percentage Change (APC), its 95% Confidence Interval (95% CI), and its p-value. Better explaining, non-statistically significant results (p-value > 0.05) indicate stability regardless of the APC value. For statistically significant results (p-value < 0.05), the APC sign indicates increasing (when positive) or decreasing (when negative) trends, and the absolute value of the APC indicates the magnitude of these trends. The 95% CI helps interpret the precision of this measure, with the lower the 95% CI, the greater the statistical strength of the result obtained, and vice versa. Furthermore, heatmaps were created by using R software 4.4.2 ®. 

## RESULTS

 Of the 36 countries in the Americas, 21 were evaluated in this research. The remaining 15 countries were excluded due to missing data for one or more years during the period analyzed, as detailed in the Methods section. 

**Figure 1:**
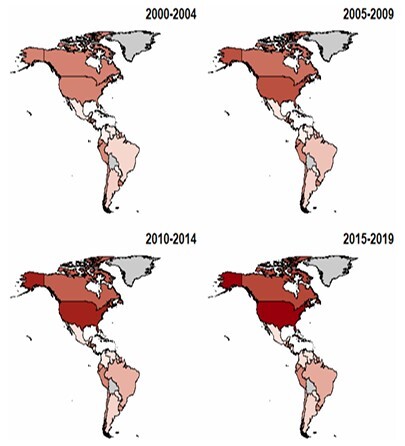
Heatmap representing the age-adjusted mortality trends by dementia in women across the American continent between 2000 and 2019.

**Figure 2:**
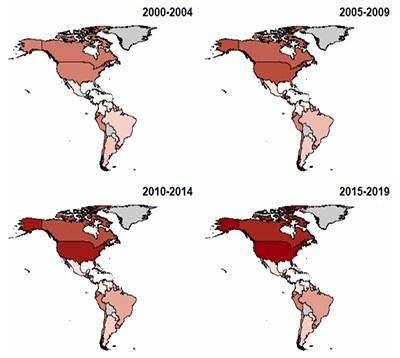
Heatmap representing the age-adjusted mortality trends by dementia in men across the American continent between 2000 and 2019

 In both sexes, the highest mortality rates were observed in the United States and Canada. Significant mortality rates were observed in Peru, Chile, Ecuador, Uruguay, Honduras, and Guatemala. At the same time, the lowest rates were observed in Colombia, Paraguay, and Venezuela. Overall, mortality rates were higher in women, with the exception of Suriname and Nicaragua, which demonstrated higher mortality in men. Figures 1 and 2 present heatmaps illustrating the evolution of dementia mortality for each sex across the American continent. 

 Although the magnitude and timing of these trends showed specific patterns for each location evaluated, general trends in dementia mortality were observed. Among the countries evaluated, Canada, the United States, Brazil, Panama, Paraguay, Peru, Nicaragua, and Suriname showed general upward trends. Meanwhile, El Salvador, Ecuador, Guyana, and Chile showed downward trends. Finally, Venezuela, Argentina, Uruguay, Belize, Honduras, Guatemala, Costa Rica, Colombia, and Cuba showed more complex trends, characterized by periods of stabilization interspersed with increases and decreases throughout the periods evaluated. A more in-depth evaluation of these results can be observed by analyzing the supplementary figures and tables presented in the following links: https://docs.google.com/document/d/12CSi8l8EZH1P0M4z6poUcvtR9O53Nr6mN8Kdc088saQ/edit?tab=t.0 and https://docs.google.com/spreadsheets/d/1dfFN9CE-kN3Z_p367Hwvq8VR3LIQmFxvxHLgynyh0Lc/edit?gid=0#gid=0. 

## DISCUSSION

 The highest dementia mortality rates were observed in the United States and Canada, while the lowest were observed in Colombia, Paraguay, and Venezuela. In most countries, mortality was consistently higher among women, although some experienced periods of narrowing gender differences. Notable exceptions include Suriname and Nicaragua, where mortality was consistently higher among men. Overall, the observed patterns were complex, with particularities in each country assessed. 

 Dementia mortality rates were highest in the United States and Canada, drive by a combination of demographic, healthcare, and diagnostic factors^7,8^. In case, these countries present highly developed healthcare systems that enable a large recognition of dementia in their population, which reduces the underreporting of it^7^. Furthermore, the high prevalence of the elderly in their population, since these countries present high life expectancies, increases the burden of dementia-related mortality^8^. Finally, environmental factors such as urbanization and greater exposure to air pollution may further exacerbate dementia risks, not only to develop it, but also to die because of it^7,8^. 

 Conversely, the lower mortality rates observed in countries like Colombia, Paraguay, and Venezuela possible reflect contrastant realities. In this perspective, dementia underdiagnoses and underreporting remain significant challenges in these locals, often driven by limited healthcare infrastructure, socioeconomic disparities, and reduced access to specialized neurological care^9^. In Venezuela, for example, the ongoing political and economic crisis has severely strained public health services, potentially contributing to inaccuracies in death certification and cause-of-death reporting^10^. Furthermore, cultural perceptions of aging and dementia in certain regions may lead to its normalization as a natural aspect of old age rather than as a medical condition warranting specific care, which may delay dementia’s recognition^11^. 

 The divergent trends across countries should also be mentioned. The observed declining rates in countries such as Ecuador and Guyana may reflect improvements in cardiovascular health, public health interventions targeting non-communicable diseases, and rising educational attainment, which collectively enhance cognitive reserve and delay dementia onset^12^. In contrast, an increasing pattern observed in countries like Brazil and Suriname may be attributed to aging populations, urban stressors, greater diagnostic precision, and possibly increased prevalence of risk factors like diabetes and obesity^13^. 

 Throughout the American continent, dementia mortality was generally higher among women, which is in accord with global observations^14,15^. As women tend to have longer life expectancy, there is a higher probability that they develop dementia and may die because of it^14^. Moreover, biological factors such as postmenopausal estrogen decline, which diminishes neuroprotective mechanisms, heighten women’s vulnerability to neurodegenerative diseases when compared with men^14^. Cultural and social dynamics may also play a role, since women traditionally bear greater caregiving burdens, which can delay recognition of their cognitive decline until more severe stages, leading to higher reported mortality^14,15^. Nonetheless, notable exceptions were observed in Suriname and Nicaragua, where men exhibited consistently higher dementia mortality^15^. These exceptions may be influenced by factors such as higher male exposure to vascular risk factors, like hypertension, diabetes, and tobacco use, and potentially differing societal patterns of healthcare access and disease reporting^15,16^. 

 From a practical perspective, these findings underscore the urgent need for tailored, multi-level interventions to address dementia mortality across the Americas^17,18^. Strengthening healthcare infrastructure, improving early detection programs, and expanding access to cognitive health services are critical measures^17^. Countries must also consider national and international collaborations to implement culturally sensitive, gender-responsive, and regionally adapted dementia care strategies^17^. Addressing social determinants of health, reducing environmental exposures, promoting cardiovascular health, and investing in public education about dementia could substantially mitigate the future burden^18^. Ultimately, a comprehensive approach that integrates biological, social, economic, environmental, and policy dimensions is essential to alter the current trajectory and improve quality of life for aging populations throughout the continent^18^. 

 As an ecological study, the results may not be directly applicable to individual-level risk factors or causative mechanisms of dementia’s mortality. Second, data accuracy relies on the quality and completeness of national reports submitted to the PAHO, which may vary between countries due to differences in surveillance systems, reporting criteria, and diagnostic capacities. Additionally, potential underreporting or misclassification of dementia deaths cannot be ruled out, particularly in regions with limited healthcare infrastructure. 

 This study presents strengths. The long study period (2000–2019) allows for an in-depth evaluation of temporal trends, offering valuable insights into changes in dementia’s mortality over time. Moreover, the use of joinpoint regression analysis provides an advanced statistical approach to detecting significant shifts in trends, allowing a more precise understanding of disease dynamics. Finally, by analyzing data across multiple countries, this study contributes to a broader epidemiological perspective on dementias, facilitating regional comparisons and informing public health policies aimed at mitigating disease burden. 

## CONCLUSIONS

 This study highlights the heterogeneous patterns of dementia-related mortality across the American continent, marked by notable sex disparities and regional variability. While demographic aging and improved surveillance partially explain the growing burden of dementia, the findings also reflect complex interplays of social, economic, cultural, and environmental determinants. 

 From a practical standpoint, these results underscore the urgent need for integrated dementia policies that align with broader aging and non-communicable disease agendas. Investment in healthcare infrastructure, professional training, community-based programs, and reliable mortality reporting systems is essential to ensure equitable management across countries with diverse capabilities. Furthermore, fostering international collaboration can help address knowledge gaps and harmonize surveillance strategies, particularly in under-resourced settings where dementia remains underrecognized. 

 Future research should prioritize the longitudinal monitoring of dementia trends using standardized methods and incorporate granular data on comorbidities, ethnicity, socioeconomic status, and geographic disparities. Cross-disciplinary approaches—bridging neurobiology, epidemiology, public health, and social sciences—will be key to developing targeted interventions that are both effective and contextually relevant. As dementia continues to rise as a global health challenge, this study contributes to building the scientific foundation needed to inform more responsive, inclusive, and sustainable solutions. 

## Data Availability

Data are derived from public domain resources. The data supporting this study’s findings are available at https://www.paho.org/en.
